# Effects of Aging Stereotype Threat on Working Self-Concepts: An Event-Related Potentials Approach

**DOI:** 10.3389/fnagi.2017.00223

**Published:** 2017-07-12

**Authors:** Baoshan Zhang, Yao Lin, Qianyun Gao, Magdalena Zawisza, Qian Kang, Xuhai Chen

**Affiliations:** ^1^School of Psychology, Shaanxi Normal University Xi’an, China; ^2^School of Psychology and Cognitive Science, East China Normal University Shanghai, China; ^3^Department of Psychology, Anglia Ruskin University Cambridge, United Kingdom

**Keywords:** aging stereotype threat, working self-concept, ERP, event related oscillations, neural underpinnings

## Abstract

Although the influence of stereotype threat (ST) on working self-concepts has been highlighted in recent years, its neural underpinnings are unclear. Notably, the aging ST, which largely influences older adults’ cognitive ability, mental and physical health, did not receive much attention. In order to investigate these issues, electroencephalogram (EEG) data were obtained from older adults during a modified Stroop task using neutral words, positive and negative self-concept words in aging ST vs. neutral control conditions. Results showed longer reaction times (RTs) for identifying colors of words under the aging ST compared to the neutral condition. More importantly, the negative self-concept elicited more positive late P300 amplitudes and enhanced theta band activities compared to the positive self-concept or neutral words under the aging ST condition, whereas no difference was found between these self-concepts and neutral words in the control condition. Furthermore, the aging ST induced smaller theta band synchronization and enhanced alpha band synchronization compared to the control condition. Moreover, we also observed valence differences in self-concepts where the negative self-concept words reduced early P150/N170 complex relative to neutral words. These findings suggest that priming ST could activate negative self-concepts as current working self-concept, and that this influence occurred during a late neural time course.

## Introduction

As an important social cognitive factor, age-related stereotypes such as physical unattractiveness, loneliness, failing performance, morbidity and lack of creativity (Rothermund and Brandtstädter, 2003; Wheeler and Berger, 2007) have been prevalent in older adults’ daily lives. Notably, negative stereotypes about cognitive deterioration among older adults are widespread (Cuddy et al., 2005). For example, many studies have revealed the stereotypic view that older adults have poor memory and are associated with stereotypic characteristics such as dementia, slow thinking and forgetfulness (McConnell, 2011). These negative stereotypes are reportedly deeply rooted in most cultures (Cuddy et al., 2005) and affect cognitive and behavioral outcomes via stereotype threat (ST; Scholl and Sabat, 2008). ST is described as the situational experience, in which stigmatized individuals feel anxious about confirming negative stereotypes pertaining to their group (Steele and Aronson, 1995). The activation of ST results in cognitive performance decrements in stereotype relevant domains (e.g., Appel and Kronberger, 2012; Tine and Gotlieb, 2013; Flore and Wicherts, 2015), in motor skills deficits (Heidrich and Chiviacowsky, 2015), and even contributes to differential health service treatment (Jones et al., 2013). Similarly, numerous studies have consistently revealed various detrimental effects of ST on older adults’ cognitive performance (e.g., Levy et al., 2012).

Meanwhile, many studies have also endeavored to illuminate the possible mechanisms underlying the effects of the aging ST on cognitive performance of the older adults (for a review, see Wheeler and Petty, 2001). A number of psychological constructs, such as beliefs about aging (Hess and Hinson, 2006), self-efficacy (Desrichard and Köpetz, 2005), poorer working memory (Mazerolle et al., 2012) and the use of prevention-focused strategies (Barber and Mather, 2013b), have been shown to mediate the relationship between the aging ST and older adults’ cognitive performance. These constructs could also contribute to the behavioral effects of aging ST.

Although numerous studies have focused on the effects of aging ST on cognitive performance (e.g., Levy et al., 2012), as well as on the possible behavioral mechanisms underlying the effects of aging ST (e.g., Wheeler and Petty, 2001), some new and very important approaches have been neglected by this literature. Two such notable omissions, which have been increasingly investigated in the context of other types of STs, concern the effects of the aging ST on working self-concept and the neural mechanisms underlying these effects (e.g., Schmader et al., 2014; Cvencek et al., 2015). Our article aims to address this gap in the current existing literature. Specifically, the current study focuses on examining the effects of the aging stereotype on working self-concept and its related neural mechanism through Event-related potentials (ERPs).

In the following sections, we begin by introducing the theoretical rationale regarding the effects of ST on working self-concept and its related neural process in older adults. Based on this literature hypotheses regarding the possible neural mechanism of effects of aging ST on working self-concept are proposed.

### Effects of Stereotype Threat on Working Self-Concept

Working self-concept refers to an active, dynamically updating part of individual self-conceptions in thought and memories (Markus and Nurius, 1986), which could influence current attitudes and predict further changes in behaviors (McConnell, 2011). As the content of working self-concept shifts rapidly according to the changing situational contexts (Kawakami et al., 2012), this context-dependent self is sensitive to the social circumstances and therefore is a more accurate predictor of behaviors than a stable, unitary self (Markus and Wurf, 1987). In addition, Wheeler et al. (2007) proposed that particular primes (e.g., stereotypes) could alter self-concepts by selectively inducing biased aspects of chronic self-concepts which are relevant to current primes. Hence, a ST situation, which is characterized by prominent situationality (Steele and Aronson, 1995), should be likely to change the accessibility of stereotype-relevant self-concepts.

Much research has demonstrated the effects of ST on the currently activated self (e.g., Schmader et al., 2014). For example, Schmader et al. (2014) found that ST shifted the activation of working self-concept process from automatic to more conscious. This resulted in the working self-concepts being more closely related to one’s conscious self-representation at that moment. In addition, when stronger math-gender stereotypes were activated, boys showed stronger math self-concepts (Cvencek et al., 2015). While much research has found that aging ST largely influenced older adults’ cognitive functioning (e.g., memory ability, Hess et al., 2004; Emile et al., 2014; Levy et al., 2014), to the authors’ best knowledge no studies paid attention to the possible effects of aging ST on working self-concept specifically. Nevertheless, given the review of previous literature pertaining to other stereotyped groups, it is reasonable to expect significant effects of the aging ST on working self- concept among older adults.

### Neural Process of Working Self-Concept and the Aging Stereotype Threat

Behavioral indicators of mental process generally, and social phenomena specifically, are criticized for their low sensitivity, high ambiguity and indirect nature. Thus, in order to understand human mental process more directly and accurately, researchers have employed more sensitive techniques from neuroscience to study neural process in social situations. In line with this approach, a wealth of studies has explored the neural processes of self-concepts and stereotyping using ERP. For example, much research has demonstrated that P300 is related to the self-concept. The P300 is thought to reflect, stimulus evaluation as well as context updating processes (Polich, 2007). P300, a positive wave with a latency of roughly 300–600 ms following target onset, was an ERP component related to attentional resource allocation (Banaschewski and Brandeis, 2007). The larger amplitude of P300 implicated grater investment of attentional resources (Debener et al., 2002). Research has found that self-related information (i.e., self-names and self-faces) elicits enhanced P300 amplitude compared to self-irrelevant stimuli, and that this implies greater engagement of attentional resources to self-related information (Tacikowski and Nowicka, 2010). Furthermore, Gray et al. (2004) have found that the P300 amplitude induced by self-relevant targets (e.g., hometown) was larger than that induced by control ones, and meanwhile more cognitive resources were allocated to self-related targets. Thus, these authors further proposed that P300 should be considered as an indicator of self-related stimuli.

Moreover, P300 has also been one of the most popular components employed in the studies on ST effects. For example, Phills et al. (2011) reported that participants trained to have stronger racial stereotyping had larger P300 amplitudes to photographs of black students than those who did not undergo such training. Similarly, Bartholow et al. (2006) indicated that P300 amplitude and latency were sensitive to racial stereotypes: counter stereotypical traits elicited larger P300 s than stereotype-consistent trials. In addition, previous studies have also shown similar effects on P300 amplitude for gender stereotypes in sentence comprehension tasks (Osterhout et al., 1997).

What patterns of neural processes relate to working self-concept in older adults subjected to the aging ST? According to Wheeler et al. (2007) that primes (e.g., stereotypes) would alter self-concepts by selectively activating a biased portion of chronic self-concepts which are relevant to current primes, it is possible that aging ST would induce negative stereotype-relevant self-concept. In addition, prior research has demonstrated that activation of a specific (e.g., stereotype-relevant) self-concept could further activate other related self-concepts, and result in a positive or negative general self-concept (Moskowitz, 2005). Hence, it is likely that aging ST would eventually activate general negative self-concept as a current working self-concept via first inducing negative aging stereotype-relevant self-concepts. That is, targets relevant to the negative self- concept should be more sensitive to ST and thus more likely to elicit enhanced brain activities. In other words, stimuli relevant to negative self-concept should elicit greater specific brain activity (i.e., larger amplitudes of relevant components) compared to unrelated stimuli when ST is primed. Therefore, if the aging ST activates negative self-concepts then self-relevant stimuli will require more attentional resources and elicit larger P300 amplitudes (Tacikowski and Nowicka, 2010). Meanwhile, in the same aging ST condition, situation-irrelevant stimuli will elicit reduced P300 than stimuli relevant to negative self-concepts.

### Characteristics of Neural Oscillations in Aging Stereotype Threat

The analysis of neural oscillations, could illuminate the neural mechanism behind the effect of the aging ST on working self-concept. Yet this valuable neuronal activation processes perspective was ignored by prior research. Traditional ERP analysis examined the cognitive processes indirectly via analyzing discrepant ERP components. However, much research has suggested that the neurophysiological basis of cognitive processes is rooted in electrical activity of nerve cells, and hence the changes of potential energy and phase for group of nerve cells were the real basis of cognitive activity (Lopes da Silva, 2006; Klimesch et al., 2007). Previous research has demonstrated that low frequency oscillation (e.g., alpha and theta band) was associated with a wide range of neural activity, whereas high frequency oscillation (e.g., gamma band) was linked to limited activation of neural assemblies in local brain (Başar et al., 2001). Although previous researchers have found stereotype-related components or self-related ERP components (e.g., P300, Tacikowski and Nowicka, 2010; Tortosa et al., 2013), they could not answer the question of how ST influenced self-concepts. This may be because the time-locked and phase-locked ERPs lost the information about the changes of power and phase in different frequency bands.

After external stimulation, functionally meaningful oscillatory electroencephalogram (EEG) responses in different frequency bands have been recorded (Başar, 1992; Pantev et al., 1994; Başar et al., 1997). Several studies identified differences in theta (~5–7 Hz) and the alpha (~9–12 Hz) frequency bands to be most sensitive to higher cognitive functioning. In relation to ERPs, it has been suggested that the P300 could reflect phase locking of evoked oscillations within theta frequency bands. Spencer and Polich’s (1999) work has shown that theta band power is sensitive to the same task variables that influence the P300 component. Research suggests that increased theta power is the main contributor to P300 (Wang and Ding, 2011). Thus, we predicted that the pattern of findings recorded using theta band power should be similar to that detected using P300. Moreover, Zhang et al. (2013) observed that early theta event-related synchronization is indicative of motivated attention: theta synchronization decreased when the participants performed distraction task. Thus, we also hypothesized that priming the aging ST will decrease theta band synchronization.

However, research using alpha band (unlike that using theta band) returns less consistent findings and tells a different story. EEG alpha activity or dominant oscillations play a key role in important processes such as alertness, perception, cognitive resource allocation, memory and emotions (Başar and Schurmann, 1996; Niedermeyer, 1997). In particular, alpha activity might direct the flow of information through the brain and allocate resources to relevant regions (Jensen and Mazaheri, 2010). Yordanova et al. (2001) have shown that during an oddball detection task, P300 response were correlated with the magnitude of an alpha power reduction. The earlier studies also reported that the simultaneously recorded P300 and alpha activity manifested a similar sensitivity to the oddball task, event-related alpha appears to be functionally associated with the cognitive processing demands eliciting P300 (Yordanova and Kolev, 1998). According to the alpha inhibition hypothesis, low alpha activity reflects active neuronal processing (Käthner et al., 2014). However, Klimesch et al. (1999) found that alpha activity was increased during encoding and retention of a working memory task and alpha synchronization when task demands (during episodic short-term memory processing) were highest. Also, Mu and Han (2010) found that self-judgments induced increased alpha synchronization relative to other-judgment at 400–600 ms. The mixed results indicate that the empirical data on the functional role of alpha band oscillations is not yet conclusive.

Based on these, to further understand the neural underpinnings of working self-concept under aging ST conditions, analysis of event related oscillations (ERO) is needed. It could provide unique information about the event-related spectral perturbation (ERSP) and inter-trial coherence (ITC) in a particular frequency band (Delorme and Makeig, 2004).

### The Present Study

The present study integrates measurement of working self-concept with the measurement of memory performance under the condition of aging ST explore the influence on working self-concepts. ST related to older adults’ memory usually undermines their memory performance. Evidence supporting this effect comes from a variety of experimental tasks (e.g., Hess et al., 2009a). Drawing on this literature, we created a condition of aging ST by adopting the “diagnostic memory abilities task” paradigm. Older adults were asked to complete a working self-concept task under the aging ST condition while EEG activity was recorded through ERPs.

Older adults were recruited as participants and their positive and negative self-concept words were first collected 1 or 2 days before ERP experiment. In the stage of the formal experiment, they were allocated to the aging ST condition or control condition randomly, and were required to complete a yes/no memory recognition task constituting of an encoding phase and a subsequent recognition test, which were separated by a Stroop task. The Stroop task consisted of participants’ positive and negative self-concept words and the randomly selected neutral words. The reaction times (RTs), accuracy and EEG activity were recorded throughout the memory recognition task and the Stroop task.

Previous studies have consistently shown that age ST can impair memory ability of older adults (Hess et al., 2003, 2009b; Andreoletti and Lachman, 2004; Chasteen et al., 2005; Hess and Hinson, 2006; Barber and Mather, 2013a,b, 2014). Therefore, memory task performance should be sensitive to the aging ST and could be an indicator of the efficiency of ST manipulation. Thus, in the present study, we used the memory task to check the manipulation of ST.

We conducted three types of data analyses. First, we analyzed the accuracy in the memory recognition test with one-way analysis of variance (ANOVA) to check the effectiveness of the manipulation. The RTs in the Stroop task were analyzed with a two-way repeated measures ANOVA where group identity served as a covariate, as group identity could influence the effect of ST to a large extent (O’Brien and Hummert, 2006).

Second, time-domain analysis of ERP data in Stroop task was conducted. The amplitudes of P300 component in the relevant time-window (300–450 ms) were analyzed. In addition to P300 components, we were also interested in the differences in the P150/N170 complex. There are two reasons for the relevance of this complex here. First, the P150/N170 complex has been associated with the processing of complex visual stimuli such as words and faces (Schendan et al., 1998; Bentin et al., 1999; Rossion et al., 2003; Joyce and Rossion, 2005; Dien, 2009). For example, it has been used as an indicator of the initial orthographic processing and the activation of sublexical units during visual word recognition (Holcomb and Grainger, 2006; Chauncey et al., 2008). Since our experimental task involved lexical processing, we expected that the P150/N170 complex may also be elicited differentially in the context of neutral words and positive and negative self-concept words. Second, P150/N170 complex has also been employed as a self-relevant component (Higashima et al., 2004; Carlson and Reinke, 2010). For example, self-relevant stimuli have reportedly elicited a reduced frontal P150 in relation to other stimuli (Geng et al., 2012). However, Shi (2016) reported that self-name elicited larger N170 amplitudes than all other names. Therefore, although we are not sure what the direction of the differences maybe we included this component in the current study on exploratory basis.

Finally, frequency-domain analysis of ERP data in the Stroop task was performed to examine the changes in neural oscillations. ERSP and ITC in theta frequency band was analyzed with repeated measures ANOVAs to further explore the neural mechanisms. As we have mentioned before, the results returned by alpha band are quite different from those returned by theta band and the empirical data on the functional role of alpha band oscillations is not yet conclusive. Therefore we have also included alpha band in the current study.

### Hypotheses

Based on above rationale, the influence of the aging ST on working self-concept, and its neural underpinnings were systematically investigated in the older adults. Specifically, following hypotheses were proposed:

Hypothesis 1: in aging ST condition word stimuli related to negative self-concept will elicit higher P300 amplitude than word stimuli related to positive self-concepts.

Hypothesis 2a: in the aging ST condition, theta band power would increase in response to negative self-concepts word.

Hypothesis 2b: the aging ST condition will elicit smaller theta band synchronization than the control condition.

Based on the previous literature we also set two non-directional (exploratory) research questions concerning the differences in the P150/N170 complex and alpha band. These are:

First, in P150/N170 complex, we expected a significant difference on self-concept valances or groups (i.e., experimental group and control group), or both.

Second, alpha band power and synchronization induced by aging stereotype condition will be different to those induced by the control condition.

## Materials and Methods

### Participant

Thirty-eight right-handed older adults (21 females, 17 males, ages 58–81, mean age= 67.29 years ±5.43 SD) were recruited by advertisements on voluntary basis. All of the participants were retired and in good health: none of the participants reported neurological or psychiatric disorders or received central-acting medication. They were randomly assigned to the aging ST vs. the control groups and each group consisted of 19 participants. No significant differences were found in terms of socio-demographic variables between the aging ST and the control groups: age, *F*_(1,2)_ = 0.576, *p* > 0.1 (*M* = 67.58, SD = 5.91 and *M* = 67.00, SD = 5.06, respectively) and education years, *F*_(1,2)_ = 1.000, *p* > 0.1 (*M* = 11.05, SD = 3.89 and *M* = 11.63, SD = 2.79, respectively).

This study was approved by the university’s Institutional Review Board and all the ethical guidelines from the Committee on Ethics of Research in Humans at each authors’ institution were followed. All participants had signed written informed consent in accordance with the Declaration of Helsinki. The protocol was approved by the university’s Institutional Review Board.

### Procedure

The process of the current experiment was mainly divided into two stages: preparatory stage and experimental stage.

#### Preparatory Stage

One or two days prior to ERP experiment, all participants were instructed to describe themselves with five words related to their strengths (positive self-concepts) and five words related to their weaknesses (negative self-concepts). Five neutral words used in previous studies were employed as neutral controls, and were matched with individuals’ self-concepts in terms of word number (i.e., all of the words consisted of two Chinese characters). In addition, participants were required to provide demographic information and complete a group identity questionnaire. The group identity scale aimed to assess older adults’ group identity and was an adaptation of a Scientific Identity Scale (Estrada et al., 2011). The scale consists of five unidimensional items, such as “*I have a strong sense of belonging to an elderly group*”, and “*I have come to think of myself as an ‘old person’*”. Participants were required to indicate the extent of their agreement with the statements on a 5-point Likert scale, ranging from 1 (*strongly disagree*) to 5 (*strongly agree*). The internal consistency was high (*α* = 0.85).

#### Experimental Stage^1^

The relevant information about ERP study phase was first provided to each participant when they entered into the laboratory. After this, participants were randomly assigned to negative aging ST condition or neutral control condition, and then were instructed to read different brief reports depending on their conditions (ST manipulation). Two brief reports were constructed to describe aging stereotypes or language sensitivity (neutral controls). The former one outlined evidence that the capacity of memory and thinking continues to decline with age, and explained that the aim of the current study was to test the capacity of memory accurately. The following is a sample excerpt from this report: “Many studies have indicated that the capacity of memory declines with age for a majority of people, such that they remember less information, required more time to memorize it, and have increasing difficulty in recall and recognition. In order to further explore memory in older adults, a memory test will be conducted following this report, which can capture accurately individuals’ memory capacity”. This negatively framed article was used to trigger aging ST. The other, neutral one, discussed the possible reasons for disappearance of languages and loss of language sensitivity, and portrayed the aim in the current study as that of measuring people’s sensitivity toward the minority languages. The following excerpt illustrates this: “Researchers have demonstrated that there were more than 5000 languages in the word, but almost 1400 of them are going to disappear. For example, more than 170 languages used to be spoken by North American Indians in the past, but nowadays most of the local people are used to speaking English, with original language sensitivity lost”. This neutral article was used in the control condition. The negative article about memory was 347 characters long, and the neutral article about language sensitivity was 346 characters in length. To ensure the participants understood the reports well, the researches read the articles aloud for them again.

Following the stereotype or neutral primes, participants were prepared for ERP experiment (e.g., electrode cream was applied). This took about 40 min. Then, the participants were required to sit 60 cm away from a computer screen and to complete a computerized yes/no recognition task consisting of an encoding phase and a subsequent recognition test, which were separated by the Stroop task. In the encoding phase, participants saw 50 words and were instructed to memorize all of them for the coming memory test. The computerized study trial consisted of a fixation cross (500 ms), the target word (2000 ms) and a blank screen (500 ms).

Second, participants were asked to complete a modified version of the original classic Stroop task (Stroop, 1935), which has been widely used to measure working self-concepts in previous research (e.g., Mikulincer et al., 2004; Zhang et al., 2011). Participants were required to press different keyboard keys depending on the color of the stimulus displayed as accurately and quickly as possible, while ignoring the meaning of the words. After manual response, the words disappeared and then a black screen was showed for 500 ms. Next a fixation point was displayed for 1 s prior to the next word. The colored words which included five positive self-concept words (e.g., *optimistic, good* and *positive*), five negative self-concept words (e.g., *failure, pessimistic* and *depressed*) and five neutral words (e.g., *leaf, bicycle* and *radio*) were presented in the center of the black screen. Each word was presented eight times in two different colors (red and blue) in random order. Hence, 240 trials were present in total. Practice trials with neutral colored words (red and blue) were conducted preceding the experiment to strengthen the association between colors of the stimuli and their specific response keys. In the current study, each participant completed a unique Stroop task composed of their own personalized self-concept words, as self-concepts differed in different individuals.

Finally, in the recognition test phase, participants were presented with 50 words from the encoding phase (‘old’ words) and 50 new words. They were instructed to indicate whether the target word was old or new. A test trial consisted of a fixation cross (500 ms), a target word which would not disappear until the manual response was completed, and a blank screen (500 ms). RTs, accuracy and EEG activity were recorded throughout the encoding phase, the Stroop task and the recognition test phase. After participants completed the memory test task, they were thanked for their participation and were reimbursed ¥80 for their participation time.

### EEG Recording and Data Analysis

All EEG records (bandpass 0.05–100 Hz, sampling frequency 500 Hz) were collected using an amplifier and the elastic EEG cap embedded with 64 Ag/AgCl electrodes based on an extended international 10–20 system (Brain Products Company, Germany). Fcz electrode was used as on-line reference whereas the average mastoid reference (average of TP9 and TP10) was derived off-line. Vertical and horizontal electrooculogram (EOG) signals were measured by two electrodes placed 2.5 cm above the right eye and 1 cm beside the canthi of left eye, respectively. Electrode impedances were kept below 5 kΩ. EEG and EOG recordings were amplified with a high cutoff of 100 Hz.

Offline data were exported to EEGLAB (Swartz Center for Computational Neuroscience, University of California San Diego, San Diego, CA, USA). EEG epochs from 200 ms preceding to 1000 ms following stimulus onset were extracted and then baseline was corrected (200 ms baseline before stimulus). All EEG data were first visually inspected for obvious artifacts such as eye movements. The data were then high pass filtered at 0.05 Hz and average referenced across all scalp electrodes. Epochs with large artifacts (exceeding ±100 μV) were removed. Independent component analysis (ICA) using the Infomax algorithm was used to obtain independent components (ICs) from scalp EEG activity and ICs representing artifacts were rejected to obtain purer EEG signals. Each EEG average consisted of about 222 epochs.

When conducting analysis of ERP data, ERP waveforms were first averaged for each condition and for each participant, and then were grand-averaged for each condition separately. According to the observation and preliminary analysis of the grand average waveforms, ERP data were exported within separate two time windows: 140–180 ms (P150/N170) and 300–450 ms (P300).

After ERP analysis, analysis of ERO was conducted, in which the convolution of sinusoidal small waves and time-domain data was executed, and wavelet cycle increased with enhancing frequency of ERP waves (Delorme and Makeig, 2004). Single-trial analysis was first conducted and then multiple trials were averaged to obtain ERSP and ITC for each condition. Next, the ERSP and ITC post-stimulus onsets were baseline corrected with the 200 ms baseline before stimulus presentation. In the current study, the data were segmented from 1000 ms pre to 2000 ms post target onset to ensure the relevant time windows (−100 ms to 800 ms) were not lost after frequency-domain analysis.

To further examine the effect of aging ST, more detail statistical analysis was conducted on RT and ERP data. First, the effects of aging stereotype conditions (negative vs. control) and self-concept valences (negative, neutral and positive) on RTs were analyzed using repeated measures ANOVA with these two independent variables entered as between-subject and within-subject factors, respectively. Next, for the ERP data, repeated ANOVAs were performed for each of the time windows with a between-subject factor of the aging stereotype conditions and within-subject factors of the self-concept valences and region (left anterior: F7, F5, F3, FT7, FC5, FC3; middle anterior: F1, FZ, F2, FCZ, FC1, FC; right anterior: F8, F6, F4, FT8, FC6, FC4; left central: T7, C5, C3, TP7, CP5, CP3; middle central: C1, CZ, C2, CP1, CP2, CPZ; right central: T8, C6, C4, TP8, CP6, CP4; left posterior: P7, P5, P3, PO7, PO3, O1; middle posterior: P1, PZ, P2, POZ, OZ; right posterior: P8, P6, P4, PO8, PO4, O2, Dien and Santuzzi, 2004). In order to find the discrepant frequency bands and time windows, data which showed statistical discrepancy between different conditions in ERSP and ITC were first exported by EEGLAB toolbox, and then the data in probably discrepant frequency bands and time windows were exported for further statistical analysis (the variables set were same as ERP data). Greenhouse-Geisser and Bonferroni corrections were used for *F*- and *p*-values respectively, when degrees of freedom were greater than 1.

## Results

### Memory Performance

A one-way ANOVA was conducted for accuracy in the yes/no memory recognition task. The aging stereotype condition served as a between-subject factor. Partial *η*^2^ (eta squared) was taken as indicator of effect size. According to Cohen’s guideline, *η*^2^ = 0.01 indicates a small effect, *η*^2^ = 0.06 a medium effect and *η*^2^ = 0.14 a large effect (Cohen, 1988). The analysis revealed that the main effect of the aging stereotype condition was significant for accuracy, *F*_(1,36)_ = 7.609, *p* < 0.05, partial *η*^2^= 0.174: accuracy was lower in the aging stereotype group (*M* = 39.28%, SD = 20.69%) than in the control group (*M* = 56.12%, SD = 16.75%), indicating that manipulation of reading excerpts had effectively primed the aging ST. This is in line with previous results which showed that ST impairs memory performance (e.g., Levy et al., 2012).

### Behavioral Results

A two-way repeated measures ANOVA was performed for RT data. Three percent RTs with wrong responses or exceeding 3SD from the mean value of each condition were removed. Results showed that only the main effect of aging stereotype conditions was marginally significant, *F*_(1,35)_ = 3.082, *p* = 0.088, partial *η*^2^ = 0.081, indicating that RTs were longer under the negative aging ST (*M* = 638.00 ms, SD = 168.30 ms) than under the control condition (*M* = 548.16 ms, SD = 132.32 ms). The main effect of self-concept valences, *F*_(2,70)_ = 1.935, *p* > 0.1, partial *η*^2^ = 0.052, and the two-way interaction of aging stereotype conditions and self-concept valences, *F*_(2,70)_ = 0.549, *p* > 0.1, partial *η*^2^ = 0.015, were both statistically nonsignificant. Further analysis showed that there was no difference between positive self-concepts (*M* = 635.68 ms, SD = 168.02 ms), negative self-concepts (*M* = 646.12 ms, SD = 175.03 ms) and neutral words (*M* = 632.18 ms, SD = 165.43 ms) in negative aging ST condition, *F*_(2,72)_ = 2.28, *p* > 0.1.

### ERP Results

#### P150/N170

**Figure 1:**
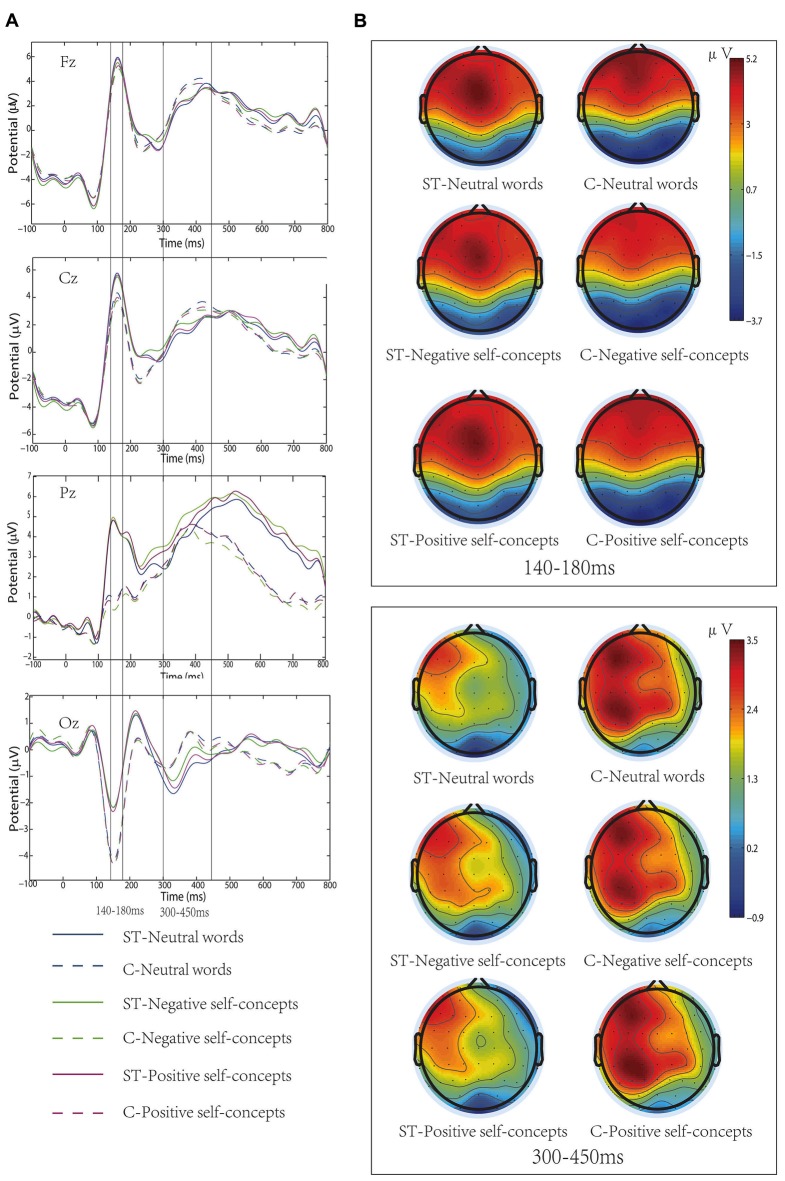
**(A)** Grand-average event-related potentials (ERPs) elicited by neutral words, positive self-concepts and negative self-concepts words at Fz, Cz, Pz and Oz areas in aging stereotype threat (ST) and control groups (C). **(B)** Topographical maps for P150/N170 (140–180 ms) and P300 (300–450 ms).

The results of an ANOVA analysis conducted at 140–180 ms interval showed a marginally significant main effect of self-concept valences, *F*_(2,72)_ = 2.598, *p* = 0.082, partial *η*^2^ = 0.067, and a significant main effect of region, *F*_(8,288)_ = 63.922, *p* < 0.05, partial *η*^2^ = 0.640. In addition, the interaction of these two variables was also significant, *F*_(16,576)_ = 2.214, *p* < 0.05, partial *η*^2^ = 0.058. Further simple effects tests indicated that neutral words elicited more positive amplitudes than negative self-concepts over left anterior, *F*_(2,74)_ = 3.861, *p* < 0.05, partial *η*^2^ = 0.094, middle anterior, *F*_(2,74)_ = 4.553, *p* < 0.05, partial *η*^2^ = 0.110, left central, *F*_(2,74)_ = 3.070, *p* < 0.05, partial *η*^2^ = 0.077 and middle central area, *F*_(2,74)_ = 4.388, *p* < 0.05, partial *η*^2^ = 0.106 (see Table 1 and Figure 1).

**Table 1:** The average P150/N170 amplitudes and standard deviations for neutral words, negative self-concepts and positive self-concepts words over different areas.

	Left anterior	Middle anterior	Left central	Middle central
	*M* (SD)	*M* (SD)	*M* (SD)	*M* (SD)
Neutral words	4.89 μV	5.39 μV	2.47 μV	3.79 μV
	(4.29 μV)	(5.24 μV)	(2.80 μV)	(4.41 μV)
Negative self-concepts	4.60 μV	5.06 μV	2.30 μV	3.52 μV
	(4.33 μV)	(5.39 μV)	(2.85 μV)	(4.58 μV)
Positive self-concepts	4.72 μV	5.22 μV	2.37 μV	3.63 μV
	(4.37 μV)	(5.37 μV)	(2.86 μV)	(4.43 μV)

#### P300

At the 300–450 ms time window, the main effect of region was significant, *F*_(8,288)_ = 9.116, *p* < 0.05, partial *η*^2^ = 0.202. And the two-way interaction of region × self-concept valences was also significant, *F*_(16,576)_ = 3.269, *p* < 0.05, partial *η*^2^ = 0.083. Further analysis revealed that both negative self-concepts and positive self-concepts elicited more positive deflection than neutral words over left central, *F*_(2,74)_ = 2.773, *p* < 0.05, partial *η*^2^ = 0.070, left posterior, *F*_(2,74)_ = 3.499, *p* < 0.05, partial *η*^2^ = 0.086 and middle posterior areas, *F*_(2,74)_ = 3.707, *p* < 0.05, partial *η*^2^ = 0.091 (see Table 2).

**Table 2:** The average P300 amplitudes and standard deviations for neutral words, negative self-concepts and positive self-concepts words over different areas.

	Left central	Left posterior	Middle posterior
	*M* (SD)	*M* (SD)	*M* (SD)
Neutral words	2.17 μV	1.27 μV	1.17 μV
	(1.54 μV)	(1.16 μV)	(1.33 μV)
Negative self-concepts	2.31 μV	1.42 μV	1.38 μV
	(1.48 μV)	(1.07 μV)	(1.28 μV)
Positive self-concepts	2.34 μV	1.42 μV	1.32 μV
	(1.62 μV)	(1.15 μV)	(1.34 μV)

In addition, the two-way interaction between aging stereotype conditions and self-concept valences was significant, *F*_(2,72)_ = 6.207, *p* < 0.05, partial *η*^2^ = 0.147. Further simple effect tests showed that there was no difference between positive self-concepts (*M* = 2.07 μV, SD = 1.46 μV), negative self-concepts (*M* = 2.02 μV, SD = 1.46 μV) and neutral words (*M* = 2.14 μV, SD = 1.44 μV) in control conditions, *F*_(2,36)_ = 1.081, *p* > 0.1, partial *η*^2^ = 0.057. Whereas negative self-concepts elicited more positive amplitudes (*M* = 1.55 μV, SD = 1.36 μV) than positive self-concepts (*M* = 1.39 μV, SD = 1.49 μV) and neutral words (*M* = 1.27 μV, SD = 1.31 μV) under negative aging ST condition, and the difference between positive self-concepts and neutral words was nonsignificant, *F*_(2,36)_ = 6.139, *p* < 0.05, partial *η*^2^ = 0.254 (see Figure 1).

The two-way interaction of region × aging stereotype conditions was nonsignificant, *F*_(8,288)_ = 0.239, *p* > 0.1, partial *η*^2^ = 0.007 as was the three-way interaction of region × self-concept valences × aging stereotype conditions, *F*_(16,576)_ = 0.774, *p* > 0.1, partial *η*^2^ = 0.021.

### ERO Analysis

#### Event Related Spectral Perturbation (ERSP)

**Figure 2:**
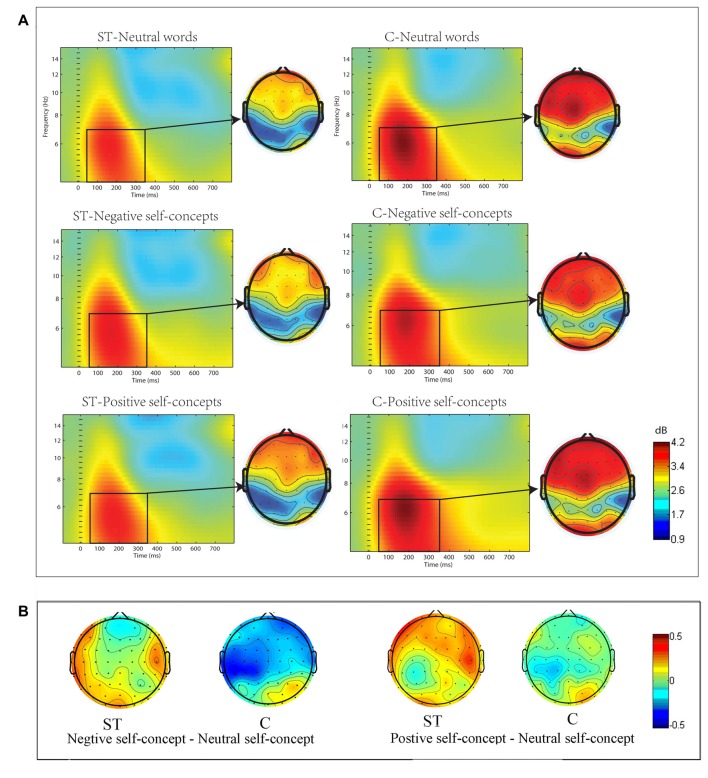
**(A)** The event-related spectral perturbation (ERSP) elicited by neutral words, positive self-concepts and negative. self-concepts at P7 (an electrode point was randomly selected to serve as the representative of the electrode points in left posterior area) in aging ST and control groups (C) were presented by square chart. Red represented enhanced power and blue represented reduced power, dB was the unit. The topographical maps on the right side of the square chart illustrate the energy distribution for 4–7 Hz at 50–350 ms for each condition. **(B)** Topographic maps of differences between different self-concept valances in control and aging stereotype condition for 4–7 Hz at 50–350 ms.

The ERSP in the theta (4–7 Hz) frequency band showed significant differences across conditions. The three-way ANOVA analysis conducted at 50–350 ms time window indicated that the main effect of region was significant, *F*_(8,272)_ = 9.932, *p* < 0.05, partial *η*^2^ = 0.226, and the three-way interaction of aging stereotype conditions × self-concept valences × region was also significant, *F*_(16,544)_ = 2.119, *p* < 0.05, partial *η*^2^ = 0.059. Further analysis revealed that the activities in theta band showed no difference between positive self-concepts (*M* = 1.13 dB, SD= 0.82 dB), negative self-concepts (*M* = 0.84 dB, SD= 0.86 dB) and neutral words (*M* = 1.02 dB, SD= 1.13 dB) in the control condition over left posterior area, *F*_(2,34)_ = 1.686, *p* > 0.1, partial *η*^2^ = 0.090, whereas negative self-concepts (*M* = 0.73 dB, SD = 1.18 dB) induced stronger power than neutral words (*M* = 0.29 dB, SD = 1.02 dB) under negative ST condition over left posterior area, *F*_(2,34)_ = 3.932, *p* < 0.05, partial *η*^2^ = 0.188 (see Figure 2).

The ERSP in the alpha frequency band showed nonsignificant differences across conditions, all Fs < 2.3, all *p*s > 0.1.

#### Inter-Trial Coherence (ITC)

**Figure 3:**
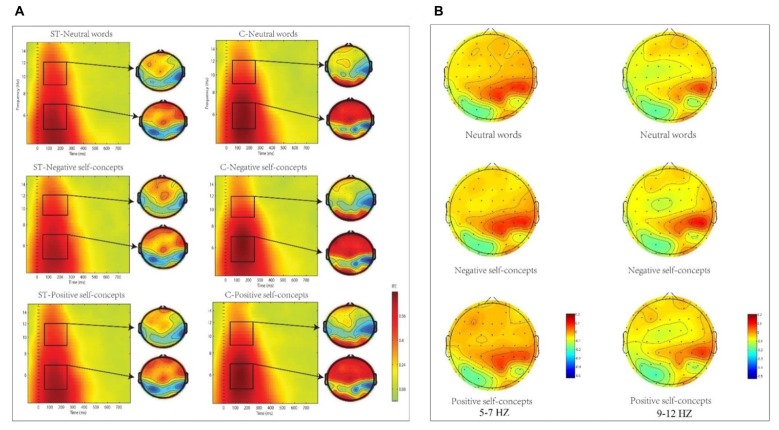
**(A)** The inter-trial coherence (ITC) elicited by neutral words, positive self-concepts and negative self-concepts at Cz in aging ST and control groups (C) were presented by square chart. Red represented enhanced ITC and blue represented reduced ITC. The topographical maps on the right side of the square chart illustrate the ITC distribution for 5–7 Hz and 9–12 Hz at 50–250 ms for each condition. **(B)** Topographic maps of condition differences for 5–7 Hz (left) and 9–12 Hz at 50–250 ms (right).

The analysis in theta (5–7 Hz) frequency band at 50–250 ms time window only found marginally significant two-way interaction of aging stereotype conditions × region, *F*_(8,272)_ = 2.096, *p* = 0.089, partial *η*^2^= 0.058, and no other significant effects were found. Further simple effect tests indicated that theta phase coherence decreased under negative aging ST condition compared to control condition. This applied to two regions: the middle central region, *F*_(1,36)_ = 6.446, *p* < 0.05, partial *η*^2^ = 0.152 (*M* = 0.11, SD = 0.03 for aging ST condition and *M* = 0.14, SD = 0.03 for control condition) and right central region, *F*_(1,36)_ = 5.772, *p* < 0.05, partial *η*^2^ = 0.138 (*M* = 0.11, SD = 0.02 foraging ST condition and *M* = 0.13, SD = 0.03 for control condition, see Figure 3).

The two-way ANOVA for alpha (9–12 Hz) ITC at 50–250 ms interval indicated that only the main effect of aging stereotype condition was significant, *F*_(1,34)_ = 4.715, *p* < 0.05, partial *η*^2^ = 0.122. Further analysis showed that higher alpha ITC was induced under negative aging ST condition (*M* = 0.11, SD = 0.02) than under the control condition (*M* = 0.10, SD = 0.01), *F*_(1,34)_ = 4.715, *p* < 0.05, partial *η*^2^ = 0.122. No other differences were statistically significant (see Figure 3).

## Discussion

The current study uniquely explored the effect of aging ST on older adults’ working self-concept using an ERP technique. The results indicated that older adults showed longer RT to all kinds of words in situation of aging stereotype. As predicated, negative self-concepts words elicited larger P300 amplitude than positive self-concepts or neutral words under situation of the aging ST, whereas no differences were found between the negative and positive self-concept words and the neutral words in the control condition. Moreover, negative self-concept words elicited smaller P150/N170 complex than neutral words in both aging ST and control conditions. Furthermore, the negative self-concepts words induced higher theta power than the neutral words in the aging ST condition, whereas there was no difference between them in the control condition. In addition, the activation of aging ST induced reduced theta band synchronization and enhanced alpha band synchronization relative to the control condition for all kinds of self-concept words and neutral words. The significance of these findings are discussed below.

### Aging Stereotype Threat Changes Current Working Self-Concepts to a Negative Self-Concept

Wheeler et al. (2007) have proposed that priming aging ST was likely to induce stereotype-relevant self-concepts. It has been widely demonstrated that self-relevant information was preferentially accessible to individuals’ attentional resources (Tacikowski and Nowicka, 2010). That is, the current, or salient, working self-concepts would engage more cognitive resource. Previous research has also found that concealing negative stereotype could change adolescents’ negative self-representation as current working self-concepts (Zhang et al., 2011). In the current study, we found that the negative self-concepts elicited larger P300 amplitudes than positive self-concepts or neutral words in the aging ST condition, and the relevant ERP components amplitudes were smaller in the aging ST condition than in the control condition. The finding that negative self-concepts elicited larger amplitudes in ST condition also suggested that the negative self-concepts allocated more cognitive resource than the positive self-concepts or neutral words. That is, aging ST resulted in the negative self-concepts becoming the current working self-concepts. In addition, we also found that the aging ST did not influence early P150/N170 complex, which suggested that the influence of the aging stereotype on older adults’ self-concepts occurred in a late neural time course. This result was consistent with the previous finding that ST had no effect on early ERP components (Wang et al., 2010).

The cognitive-relevant neuropsychological paradigms were always associated with strong evoked theta activity (e.g., Choi et al., 2010). Previous research has suggested that increased early theta power primarily reflected the activation of neural networks involved in allocation of cognitive resource related to target stimuli (Missonnier et al., 2006). In the current study, negative self-concepts induced larger theta power than neutral words under the aging ST condition, which suggested that negative self-concepts were allocated more attentional resources. Unlike expectation, there were no significant differences in the alpha band power between the two conditions. This is perhaps due to the fact that alpha band in the EEG can be influenced by workload, fatigue and some environmental factors (van Erp et al., 2010; Käthner et al., 2014).

### Aging Stereotype Threat Depleted Task-Relevant Cognitive Resource

Previous research has demonstrated that ST influences the allocation of cognitive resources (Sherman et al., 2005; Allen et al., 2009). Popham and Hess (2015) proposed that young adults under ST experienced decreased availability of cognitive resources, as indexed by relatively poor performance on a test of working memory. In addition, Dembo and Eaton (1997) also proposed that ST depletes cognitive resources. Our electrophysiological results were also consistent with this previous research. We found that the aging ST elicited reduced theta band synchronization and enhanced alpha band synchronization compared to neutral controls at 50–250 ms time window. Previous research has suggested that more extensive distraction which using a top-down cognitive resource allocation was associated with reduced theta synchronization (Zhang et al., 2013). In addition, alpha synchronization to reflect an active cognitive process. Taken together, as reduced theta band synchronization and enhanced alpha band synchronization here were found in ST condition, it is possible that aging stereotype depleted task-relevant cognitive resource in our study.

### Implications and Limitations

The majority of previous studies have explored the association between gender or racial stereotypes and specific self-concepts (e.g., academic self-concepts, Cvencek et al., 2015). Although aging stereotypes influence older adults to a large extent (Sánchez Palacios et al., 2009), little research has focused on the effect of aging ST on older adults’ self-concepts. The present study extended current theoretical knowledge on the self-concept via examining the influence of aging ST on older adults’ working self-concepts and their neural underpinnings. In addition, the current study also has practical implications for coping with the aging ST. Previous research has found that once activated the self-concepts could direct subsequent behavior (Wheeler et al., 2007). Our findings show that older adults who experience aging ST activate their negative self-concepts. Activation of such self-concept is reportedly associated with anxiety and depressive symptoms (Verplanken et al., 2007). Hence, more attention must be afforded to helping older adults combat the effects of the aging ST. In the light of our study this could be achieved by training older adults’ working self-concept to become positive before entering a ST situation.

However, some limitations of the current study should be taken in to consideration. First, our behavioral data showed that only the main effect of aging stereotype condition was marginally significant. This result indicates that behavioral data do not support the notion that priming aging ST could activate the working self-concept—a finding which is inconsistent with previous studies (Schmader et al., 2014; Cvencek et al., 2015). One possible reason is that behavioral data may not be sensitive enough. Previous studies have shown that one advantage of EEG over behavioral measures is that they can provide a measure of processing of stimuli even when there is no behavioral change (Luck, 2005). Another reason may be insufficient sensitivity of the working self-concept measure used in this study (STROOP color naming task) especially given the rather small sample size (for behavioral researches). Both factors may have contributed to the nonsignificant results. Future research should use a more sensitive measure of working self-concept to rule this possibility out. Then, compared with behavioral procedures, ERPs provide a continuous measure of processing between a stimulus and a response, making it possible to determine which stage(s) are being affected by a specific experimental manipulation. Therefore, we pay more attention to the neural results. However, relatively low spatial resolution of ERP technique did not allow for answering the question of which cerebral areas and neural networks were relevant to the influence of ST on working self-concepts. Hence, the functional magnetic resonance imaging (fMRI) studies should be conducted to investigate this issue further. Second, as stereotype could be activated explicitly or implicitly (Smith and White, 2002), and these two activation modes are quite different: the explicit mode makes the relevance of an aging stereotype more salient, and is more detrimental, whereas the implicit mode makes the stereotype easier to ignore (Shih et al., 1999). The current study only took the explicit mode into account. Further research could fruitfully explore how implicit aging stereotypes influence working self-concepts. Finally, the roles of socioeconomic status, IQ and memory ability were largely ignored in the present study. Although participants were randomly assigned to our experimental conditions, which should theoretically control for differences in socioeconomic status, IQ and memory ability, there is a small chance that participants across conditions may nonetheless have varied with respect to these variables. This could, in turn, has affected the accuracy of the memory task, and, by extension, our judgment of ST manipulation. Future studies should fully consider these variables and use scales to measure them (for example, use MMSE to measure the level of cognitive impairment of participants) to record more comprehensive information about participants.

## Conclusion

The current study explored the effect of aging ST on older adults’ working self-concept. This study demonstrated that priming aging ST could activate negative self-concepts as current working self-concept and that this influence occurred during a late neural time course as indicated by ERPs and ERO measurements. Moreover, our results confirm that aging ST depleted task-relevant cognitive resource.

## Author Contributions

BZ conceived the study, designed the protocol and prepared the manuscript. YL helped to analyze the data and prepare the manuscript and gave significant comments on the manuscript. QG carried out the study design, analyzed the data and helped to prepare the manuscript. MZ helped to deal with language issue and gave his significant comments for improving the manuscript. QK revised the manuscript critically. XC helped to design the protocol and draft the manuscript, and gave his significant comments for improving the manuscript. All authors have read and approved the final version of the manuscript.

## Conflict of Interest Statement

The authors declare that the research was conducted in the absence of any commercial or financial relationships that could be construed as a potential conflict of interest.

## Footnotes

^1^In order to ensure authenticity of the experimental conditions, a lexical recognition task was included in the procedure in line with previous researches. Participants were first asked to learn words, and then to complete a Stroop task, and finally a recognition test was conducted.
